# *N*-(2-Furylcarbon­yl)piperidine-1-carbo­thio­amide

**DOI:** 10.1107/S1600536808020977

**Published:** 2008-07-12

**Authors:** J. Duque, O. Estévez-Hernández, Yvonne Mascarenhas, J. Ellena, Rodrigo S. Corrêa

**Affiliations:** a Department of Structure Analysis, Institute of Materials, University of Havana, Cuba; b Grupo de Cristalografía, Instituto de Física de São Carlos, Universidade de São Paulo, São Carlos, Brazil

## Abstract

The title compound, C_11_H_14_N_2_O_2_S, was synthesized from furoyl isothio­cyanate and piperidine in dry acetone. The thio­urea group is in the thio­amide form. The thio­urea group makes a dihedral angle of 53.9 (1)° with the furan carbonyl group. In the crystal structure, mol­ecules are linked by inter­molecular N—H⋯O hydrogen bonds, forming one-dimensional chains along the *c* axis. An intramolecular N—H⋯O hydrogen bond is also present.

## Related literature

For general background, see: Aly *et al.* (2007 ▶); Estévez-Hernández *et al.* (2006 ▶, 2007 ▶); Koch (2001 ▶). For related structures, see: Dago *et al.* (1987 ▶); Plutin *et al.* (2000 ▶); Pérez *et al.* (2008 ▶); Duque *et al.* (2008 ▶). For the synthesis, see: Otazo-Sánchez *et al.* (2001 ▶).
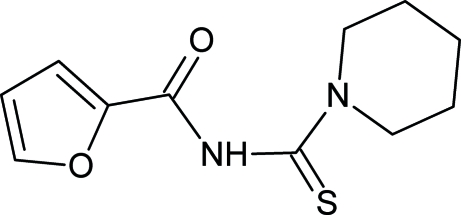


## Experimental

### 

#### Crystal data

C_11_H_14_N_2_O_2_S
*M* *_r_* = 238.3
Orthorhombic, 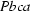

*a* = 31.6377 (15) Å
*b* = 8.6787 (4) Å
*c* = 8.5308 (3) Å
*V* = 2342.34 (18) Å^3^
*Z* = 8
Mo *K*α radiation
μ = 0.26 mm^−1^
*T* = 294 K
0.15 × 0.13 × 0.06 mm

#### Data collection

Nonius KappaCCD diffractometer
Absorption correction: none
4308 measured reflections
2387 independent reflections
1550 reflections with *I* > 2σ(*I*)
*R*_int_ = 0.039

#### Refinement

*R*[*F* ^2^ > 2σ(*F* ^2^)] = 0.067
*wR*(*F* ^2^) = 0.205
*S* = 1.10
2387 reflections
145 parameters
H-atom parameters constrained
Δρ_max_ = 0.35 e Å^−3^
Δρ_min_ = −0.35 e Å^−3^

### 

Data collection: *COLLECT* (Enraf–Nonius, 2000 ▶); cell refinement: *SCALEPACK* (Otwinowski & Minor, 1997 ▶); data reduction: *DENZO* (Otwinowski & Minor, 1997 ▶) and *SCALEPACK*; program(s) used to solve structure: *SHELXS97* (Sheldrick, 2008 ▶); program(s) used to refine structure: *SHELXL97* (Sheldrick, 2008 ▶); molecular graphics: *ORTEP-3 for Windows* (Farrugia, 1997 ▶); software used to prepare material for publication: *WinGX* (Farrugia, 1999 ▶).

## Supplementary Material

Crystal structure: contains datablocks global, I. DOI: 10.1107/S1600536808020977/ww2121sup1.cif

Structure factors: contains datablocks I. DOI: 10.1107/S1600536808020977/ww2121Isup2.hkl

Additional supplementary materials:  crystallographic information; 3D view; checkCIF report

## Figures and Tables

**Table 1:** Hydrogen-bond geometry (Å, °)

*D*—H⋯*A*	*D*—H	H⋯*A*	*D*⋯*A*	*D*—H⋯*A*
N1—H1⋯O2	0.86	2.38	2.756 (3)	107
N1—H1⋯O1^i^	0.86	2.18	2.994 (4)	157

Symmetry code: (i) 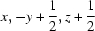.

**Fig. 1.**
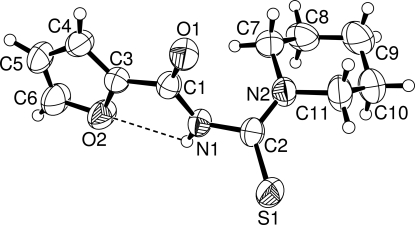
View of the molecular structure of the title compound (50% probability displacement ellipsoids). Intramolecular Hydrogen bonds (N1–H1···O2) are shown as dashed lines.

**Fig. 2.**
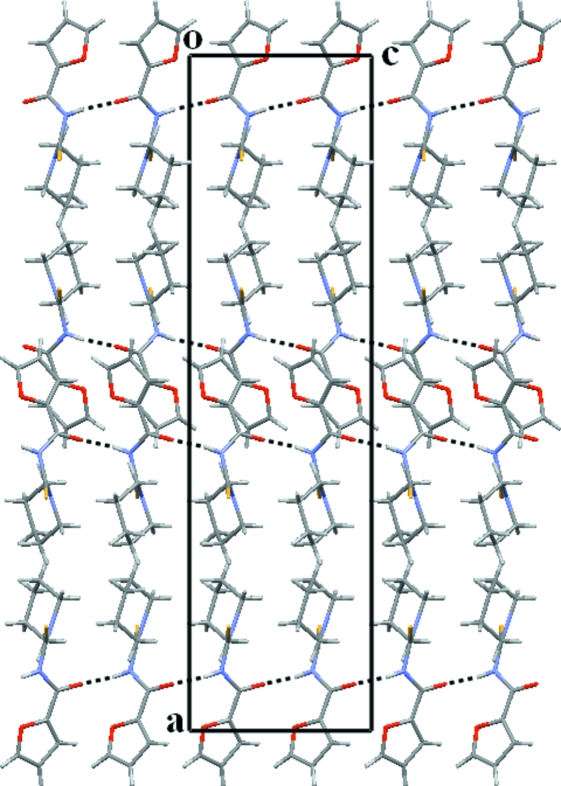
View of the crystal packing of the title compound projected down the b axis. Intermolecular hydrogen bonds (N1–H1···O1) form one-dimensional chains along c axis. The hydrogen bonds are shown as dotted lines.

## supplementary crystallographic information

### Comment

Thiourea and its derivatives form a versatile family of ligands that are suitable to form complexes with ions of transition and post-transition metal through the S atom (Koch *et al.*, 2001; Aly *et al.*, 2007). The title compound shows outstanding complexation properties (Estévez-Hernández *et al.*, 2006). The potential applications of this class of ligands as ionophores or chemical modifiers in amperometric sensors (Estévez-Hernández *et al.*, 2007) have stimulated our interest in their crystal structure. The title compound crystallizes in the thioamide form. The main bond lengths and torsion angles are within the ranges obtained for similar compounds (Dago *et al.*, 1987; Plutin *et al.*, 2000). All the C–N bonds of thiourea fragment C1–N1, C2–N1 and C2–N2 (Table1) are in the range 1.415 (4)–1.327 (4) Å, intermediate between those expected for single and double C–N bonds (1.47 and 1.27 Å respectively). These results can be explained by the existence of resonance in this part of molecule (Pérez *et al.*, 2008; Duque *et al.*, 2008). The central thiourea fragment (N1—C2—S1—N2) makes dihedral angle of 53.9 (1)° with the furan carbonyl (C1—C3—C4—C5—C6—O2) group. The *trans*-*cis* geometry in the thiourea moiety is stabilized by the N1–H1···O2 intramolecular hydrogen bond (Fig.1 and Table 2). In the crystal structure symmetry related molecules are linked by N1–H1···O1 intermolecular hydrogen bonds to form one-dimensional chains along *c* axis (Figs. 2 and Table 2).

### Experimental

The title compound was synthesized according to a previous report (Otazo-Sánchez *et al.*, 2001), by converting furoyl choride into furoyl isothiocyanate and then condensing with piperidine. The resulting solid product was crystallized from ethanol yielding X-ray quality single crystals (m.p 120–121°C). Elemental analysis (%) for C_11_H_14_N_2_O_2_S calculated: C 55.46, H 5.88, N 11.76, S 13.45; found: C 55.23, H 5.90, N 11.63, S 13.32.

### Refinement

All H atoms were refined with *U*_iso_(H)=1.2*U*_eq_(C/N).

### Crystal data

**Table:**

C_11_H_14_N_2_O_2_S_1_	*F*_000_ = 1008
*M**_r_* = 238.3	*D*_x_ = 1.352 Mg m^−^^3^
Orthorhombic, *P**b**c**a*	Mo *K*α radiation λ = 0.71073 Å
Hall symbol: -P 2ac 2ab	Cell parameters from 2684 reflections
*a* = 31.6377 (15) Å	θ = 2.9–26.4º
*b* = 8.6787 (4) Å	µ = 0.26 mm^−^^1^
*c* = 8.5308 (3) Å	*T* = 294 K
*V* = 2342.34 (18) Å^3^	Prism, colourless
*Z* = 8	0.15 × 0.13 × 0.06 mm

### Data collection

**Table:**

Nonius KappaCCD diffractometer	*R*_int_ = 0.039
CCD rotation images, thick slices scans	θ_max_ = 26.4º
Absorption correction: none	θ_min_ = 3.4º
4308 measured reflections	*h* = −39→39
2387 independent reflections	*k* = −10→10
1550 reflections with *I* > 2σ(*I*)	*l* = −10→10

### Refinement

**Table:**

Refinement on *F*^2^	H-atom parameters constrained
Least-squares matrix: full	*w* = 1/[σ^2^(*F*_o_^2^) + (0.1017*P*)^2^ + 1.0533*P*] where *P* = (*F*_o_^2^ + 2*F*_c_^2^)/3
*R*[*F*^2^ > 2σ(*F*^2^)] = 0.067	(Δ/σ)_max_ < 0.001
*wR*(*F*^2^) = 0.205	Δρ_max_ = 0.35 e Å^−^^3^
*S* = 1.11	Δρ_min_ = −0.35 e Å^−^^3^
2387 reflections	Extinction correction: none
145 parameters	

### Special details

**Table:**

Geometry. All e.s.d.'s (except the e.s.d. in the dihedral angle between two l.s. planes) are estimated using the full covariance matrix. The cell e.s.d.'s are taken into account individually in the estimation of e.s.d.'s in distances, angles and torsion angles; correlations between e.s.d.'s in cell parameters are only used when they are defined by crystal symmetry. An approximate (isotropic) treatment of cell e.s.d.'s is used for estimating e.s.d.'s involving l.s. planes.

### Fractional atomic coordinates and isotropic or equivalent isotropic displacement parameters (Å^2^)

**Table:**

	*x*	*y*	*z*	*U*_iso_*/*U*_eq_	
C1	0.05964 (9)	0.3587 (4)	0.2251 (4)	0.0455 (7)	
C2	0.13205 (9)	0.2817 (4)	0.2882 (3)	0.0456 (8)	
C3	0.01610 (9)	0.3667 (3)	0.2812 (3)	0.0442 (7)	
C4	−0.01794 (10)	0.4368 (4)	0.2179 (4)	0.0561 (9)	
H4	−0.0191	0.4904	0.1237	0.067*	
C5	−0.05161 (11)	0.4118 (4)	0.3246 (5)	0.0678 (11)	
H5	−0.0792	0.4468	0.314	0.081*	
C6	−0.03631 (12)	0.3293 (5)	0.4421 (5)	0.0776 (12)	
H6	−0.0521	0.2957	0.5275	0.093*	
C7	0.13358 (12)	0.5686 (4)	0.2859 (5)	0.0627 (10)	
H7A	0.1051	0.5591	0.3268	0.075*	
H7B	0.1321	0.6226	0.1865	0.075*	
C8	0.16045 (13)	0.6593 (4)	0.3996 (5)	0.0743 (11)	
H8A	0.1586	0.6123	0.5026	0.089*	
H8B	0.1495	0.7633	0.4075	0.089*	
C9	0.20640 (14)	0.6656 (5)	0.3497 (6)	0.0890 (13)	
H9A	0.209	0.7254	0.2541	0.107*	
H9B	0.223	0.7157	0.4305	0.107*	
C10	0.22286 (12)	0.5045 (5)	0.3226 (6)	0.0849 (13)	
H10A	0.2515	0.5098	0.2827	0.102*	
H10B	0.2235	0.4492	0.4213	0.102*	
C11	0.19565 (11)	0.4186 (5)	0.2081 (5)	0.0703 (11)	
H11A	0.197	0.4688	0.1065	0.084*	
H11B	0.2062	0.3143	0.1962	0.084*	
N1	0.08854 (7)	0.2942 (3)	0.3243 (3)	0.0458 (7)	
H1	0.0799	0.2594	0.4131	0.055*	
N2	0.15184 (8)	0.4140 (3)	0.2619 (3)	0.0527 (7)	
O1	0.06909 (7)	0.4111 (3)	0.0970 (2)	0.0569 (7)	
O2	0.00546 (7)	0.3001 (3)	0.4214 (3)	0.0651 (7)	
S1	0.15400 (3)	0.10814 (10)	0.28587 (13)	0.0661 (4)	

### Atomic displacement parameters (Å^2^)

**Table:**

	*U*^11^	*U*^22^	*U*^33^	*U*^12^	*U*^13^	*U*^23^
C1	0.0424 (16)	0.0478 (17)	0.0462 (19)	0.0006 (13)	−0.0034 (13)	−0.0070 (15)
C2	0.0397 (15)	0.056 (2)	0.0412 (17)	−0.0007 (14)	−0.0028 (13)	−0.0054 (15)
C3	0.0435 (16)	0.0484 (17)	0.0406 (16)	0.0015 (13)	−0.0001 (13)	0.0006 (14)
C4	0.0547 (19)	0.0541 (19)	0.060 (2)	0.0076 (16)	−0.0086 (16)	0.0046 (17)
C5	0.0423 (18)	0.070 (2)	0.091 (3)	0.0139 (17)	0.0037 (18)	−0.004 (2)
C6	0.050 (2)	0.092 (3)	0.090 (3)	0.018 (2)	0.023 (2)	0.017 (2)
C7	0.057 (2)	0.049 (2)	0.082 (3)	0.0016 (16)	0.0006 (18)	0.0000 (18)
C8	0.089 (3)	0.048 (2)	0.086 (3)	−0.0092 (19)	−0.006 (2)	−0.005 (2)
C9	0.082 (3)	0.074 (3)	0.111 (3)	−0.027 (2)	−0.018 (3)	0.002 (3)
C10	0.053 (2)	0.084 (3)	0.118 (4)	−0.017 (2)	−0.011 (2)	0.009 (3)
C11	0.0438 (19)	0.079 (3)	0.088 (3)	−0.0093 (18)	0.0112 (18)	−0.006 (2)
N1	0.0374 (13)	0.0569 (17)	0.0430 (14)	0.0020 (11)	0.0016 (10)	0.0006 (12)
N2	0.0419 (15)	0.0509 (16)	0.0655 (18)	−0.0043 (12)	0.0023 (12)	−0.0071 (13)
O1	0.0537 (13)	0.0760 (17)	0.0411 (13)	−0.0015 (11)	0.0017 (10)	0.0051 (11)
O2	0.0520 (13)	0.0813 (18)	0.0619 (15)	0.0160 (12)	0.0121 (11)	0.0164 (13)
S1	0.0485 (5)	0.0536 (6)	0.0962 (8)	0.0069 (4)	−0.0001 (4)	−0.0080 (5)

### Geometric parameters (Å, °)

**Table:**

C1—O1	1.221 (4)	C7—H7A	0.97
C1—N1	1.366 (4)	C7—H7B	0.97
C1—C3	1.460 (4)	C8—C9	1.516 (6)
C2—N2	1.327 (4)	C8—H8A	0.97
C2—N1	1.415 (4)	C8—H8B	0.97
C2—S1	1.659 (3)	C9—C10	1.510 (7)
C3—C4	1.349 (4)	C9—H9A	0.97
C3—O2	1.371 (4)	C9—H9B	0.97
C4—C5	1.418 (5)	C10—C11	1.500 (5)
C4—H4	0.93	C10—H10A	0.97
C5—C6	1.323 (5)	C10—H10B	0.97
C5—H5	0.93	C11—N2	1.461 (4)
C6—O2	1.357 (4)	C11—H11A	0.97
C6—H6	0.93	C11—H11B	0.97
C7—N2	1.475 (4)	N1—H1	0.86
C7—C8	1.511 (5)		
			
O1—C1—N1	122.9 (3)	C9—C8—H8B	109.2
O1—C1—C3	120.4 (3)	H8A—C8—H8B	107.9
N1—C1—C3	116.6 (3)	C10—C9—C8	109.9 (3)
N2—C2—N1	115.5 (3)	C10—C9—H9A	109.7
N2—C2—S1	125.9 (2)	C8—C9—H9A	109.7
N1—C2—S1	118.6 (2)	C10—C9—H9B	109.7
C4—C3—O2	110.1 (3)	C8—C9—H9B	109.7
C4—C3—C1	130.1 (3)	H9A—C9—H9B	108.2
O2—C3—C1	119.8 (3)	C11—C10—C9	111.2 (3)
C3—C4—C5	105.9 (3)	C11—C10—H10A	109.4
C3—C4—H4	127	C9—C10—H10A	109.4
C5—C4—H4	127	C11—C10—H10B	109.4
C6—C5—C4	107.1 (3)	C9—C10—H10B	109.4
C6—C5—H5	126.5	H10A—C10—H10B	108
C4—C5—H5	126.5	N2—C11—C10	110.7 (3)
C5—C6—O2	111.0 (3)	N2—C11—H11A	109.5
C5—C6—H6	124.5	C10—C11—H11A	109.5
O2—C6—H6	124.5	N2—C11—H11B	109.5
N2—C7—C8	110.0 (3)	C10—C11—H11B	109.5
N2—C7—H7A	109.7	H11A—C11—H11B	108.1
C8—C7—H7A	109.7	C1—N1—C2	123.2 (3)
N2—C7—H7B	109.7	C1—N1—H1	118.4
C8—C7—H7B	109.7	C2—N1—H1	118.4
H7A—C7—H7B	108.2	C2—N2—C11	121.6 (3)
C7—C8—C9	112.2 (4)	C2—N2—C7	125.4 (3)
C7—C8—H8A	109.2	C11—N2—C7	113.0 (3)
C9—C8—H8A	109.2	C6—O2—C3	105.9 (3)
C7—C8—H8B	109.2		
			
O1—C1—C3—C4	−5.9 (5)	N2—C2—N1—C1	59.9 (4)
N1—C1—C3—C4	172.5 (3)	S1—C2—N1—C1	−121.4 (3)
O1—C1—C3—O2	176.5 (3)	N1—C2—N2—C11	−173.8 (3)
N1—C1—C3—O2	−5.2 (4)	S1—C2—N2—C11	7.6 (5)
O2—C3—C4—C5	−0.2 (4)	N1—C2—N2—C7	7.8 (4)
C1—C3—C4—C5	−178.0 (3)	S1—C2—N2—C7	−170.8 (3)
C3—C4—C5—C6	−0.5 (5)	C10—C11—N2—C2	−120.6 (4)
C4—C5—C6—O2	1.0 (5)	C10—C11—N2—C7	58.1 (4)
N2—C7—C8—C9	54.0 (5)	C8—C7—N2—C2	122.3 (4)
C7—C8—C9—C10	−53.7 (5)	C8—C7—N2—C11	−56.3 (4)
C8—C9—C10—C11	54.5 (5)	C5—C6—O2—C3	−1.1 (5)
C9—C10—C11—N2	−56.8 (5)	C4—C3—O2—C6	0.8 (4)
O1—C1—N1—C2	−0.1 (5)	C1—C3—O2—C6	178.8 (3)
C3—C1—N1—C2	−178.4 (3)		

### Hydrogen-bond geometry (Å, °)

**Table:**

*D*—H···*A*	*D*—H	H···*A*	*D*···*A*	*D*—H···*A*
N1—H1···O2	0.86	2.38	2.756 (3)	107
N1—H1···O1^i^	0.86	2.18	2.994 (4)	157

Symmetry codes: (i) *x*, −*y*+1/2, *z*+1/2.

## References

[bb1] Aly, A. A., Ahmed, E. K., El-Mokadem, K. M. & Hegazy, M. E. F. (2007). *J. Sulfur Chem.* **28**, 73–93.

[bb2] Dago, A., Simonov, M. A., Pobedimskaya, E. A., Martin, A. & Macías, A. (1987). *Kristallografiya*, **32**, 1024–1026.

[bb3] Duque, J., Estevez-Hernandez, O., Reguera, E., Corrêa, R. S. & Gutierrez Maria, P. (2008). *Acta Cryst.* E**64**, o1068. 10.1107/S1600536808012208 PMC2961410 21202587

[bb4] Enraf–Nonius (2000). *COLLECT* Enraf–Nonius BV, Delft, The Netherlands.

[bb5] Estévez-Hernández, O., Naranjo-Rodríguez, I., Hidalgo-Hidalgo de Cisneros, J. L. & Reguera, E. (2007). *Sens. Actuators B*, **123**, 488–494.

[bb6] Estévez-Hernández, O., Otazo-Sánchez, E., Hidalgo-Hidalgo de Cisneros, J. L., Naranjo-Rodríguez, I. & Reguera, E. (2006). *Spectrochim. Acta A*, **64**, 961–971. 10.1016/j.saa.2005.09.005 16330247

[bb7] Farrugia, L. J. (1997). *J. Appl. Cryst.* **30**, 565.

[bb8] Farrugia, L. J. (1999). *J. Appl. Cryst.* **32**, 837–838.

[bb9] Koch, K. R. (2001). *Coord. Chem. Rev.* **216–217**, 473–488.

[bb10] Otazo-Sánchez, E., Pérez-Marín, L., Estévez-Hernández, O., Rojas-Lima, S. & Alonso-Chamorro, J. (2001). *J. Chem. Soc. Perkin Trans. 2*, pp. 2211–2218.

[bb11] Otwinowski, Z. & Minor, W. (1997). *Methods in Enzymology*, Vol. 276, *Macromolecular Crystallography*, Part A, edited by C. W. Carter Jr & R. M. Sweet, pp. 307–326. New York: Academic Press.

[bb12] Pérez, H., Mascarenhas, Y., Estévez-Hernández, O., Santos, S. Jr & Duque, J. (2008). *Acta Cryst.* E**64**, o695–695. 10.1107/S1600536808006181 PMC2960941 21202087

[bb13] Plutin, A. M., Marquez, H., Ochoa, E., Morales, M., Sosa, M., Moran, L., Rodíguez, Y., Suarez, M., Martín, N. & Seoane, C. (2000). *Tetrahedron*, **56**, 1533–1539.

[bb14] Sheldrick, G. M. (2008). *Acta Cryst.* A**64**, 112–122. 10.1107/S0108767307043930 18156677

